# Study of CFD-DEM on the Impact of the Rolling Friction Coefficient on Deposition of Lignin Particles in a Single Ceramic Membrane Pore

**DOI:** 10.3390/membranes13040382

**Published:** 2023-03-27

**Authors:** Hao Wang, Xinyuanrui Wang, Yongping Wu, Song Wang, Junfei Wu, Ping Fu, Yang Li

**Affiliations:** 1 College of Electromechanical Engineering, Qingdao University of Science and Technology, Qingdao 266061, China; 2 Dongyue Group, Zibo 256401, China

**Keywords:** lignin, ceramic membrane, CFD-DEM, rolling friction coefficient, coordination number, porosity

## Abstract

The discrete element method coupled with the computational fluid dynamic (CFD-DEM) method is effective for studying the micro-flow process of lignin particles in ceramic membranes. Lignin particles may exhibit various shapes in industry, so it is difficult to model their real shapes in CFD-DEM coupled solutions. Meanwhile, the solution of non-spherical particles requires a very small time-step, which significantly lowers the computational efficiency. Based on this, we proposed a method to simplify the shape of lignin particles into spheres. However, the rolling friction coefficient during the replacement was hard to be obtained. Therefore, the CFD-DEM method was employed to simulate the deposition of lignin particles on a ceramic membrane. Impacts of the rolling friction coefficient on the deposition morphology of the lignin particles were analyzed. The coordination number and porosity of the lignin particles after deposition were calculated, based on which the rolling friction coefficient was calibrated. The results indicated that the deposition morphology, coordination number, and porosity of the lignin particles can be significantly affected by the rolling friction coefficient and slightly influenced by that between the lignin particles and membranes. When the rolling friction coefficient among different particles increased from 0.1 to 3.0, the average coordination number decreased from 3.96 to 2.73, and the porosity increased from 0.65 to 0.73. Besides, when the rolling friction coefficient among the lignin particles was set to 0.6–2.4, the spherical lignin particles could replace the non-spherical particles.

## 1. Introduction

With its rapid development, the papermaking industry has become a pillar in various countries [1]. However, it discharges a large amount of pulping waste liquor, resulting in environmental pollution and waste of resources. Pulping waste liquor is produced in the pulping and cooking stage and is the main pollution source of the papermaking industry. Due to its dark brown color, high viscosity, and stench, it is called black liquor [2,3,4,5]. As a representative organic matter in black liquor, lignin is not only a biomass energy with great utilization value, but also the only non-petroleum resource in nature that can provide renewable aryl compounds [6]. In the current context of a forest-based circular bioeconomy, lignin is of considerable commercial value, accounting for approximately 24–47% of the current pulp production revenue [7]. Therefore, an efficient and low-loss separation of lignin from black liquor is of great significance to alleviate environmental pollution and improve waste recycling.

The commonly used lignin separation methods include acid precipitation and membrane separation methods [8]. The acid precipitation method is characterized by a simple process and a low cost, but it requires a quantity of acid, which easily causes secondary pollution. Currently, the membrane separation technologies have made a figure in lignin separation due to its low pollution and high efficiency. Ceramic membranes present excellent thermal, chemical, and mechanical stabilities, long service life, high separation efficiency, and high mechanical strength [9,10,11]. Therefore, they have been well accepted as one of the most effective materials for lignin separation [12]. However, fouling in the filtration has become a key factor hindering the continuous industrial production of ceramic membranes, greatly reducing the lignin separation from black liquor. Therefore, the micro filtration mechanisms have attracted increasing attention, such as the movement of lignin particles, sedimentation of lignin particles on the membrane surface, and formation of filter cake. Although the visualization of micropore blockage has been rapidly developed [13], dynamic tracking of the movement and capture of lignin particles can’t be achieved due to the limitations of current experimental conditions and observation techniques. Meanwhile, the size of lignin particles is generally micron-level, and the experimental results cannot provide microscopic information on the interaction among lignin particles and that between lignin particles and fluid. Therefore, the discrete element method coupled with the computational fluid dynamic (CFD-DEM) has been proposed to investigate the dynamic characteristics of lignin particles and the formation mechanism of filter cake during filtration of ceramic membranes [14,15,16,17].

The CFD-DEM solution is a relatively new method that can dynamically characterize the particle trajectory, deposition morphology, interaction between particles and fluid, and collision among particles during filtration [18]. Therefore, it is extensively applied in the filtration and separation field. Deshpande et al. [19] adopted the DEM and CFD-DEM methods to investigate the precipitation and consolidation of packed bed/filter cake formed by monodisperse and bidisperse spherical particles under different flow conditions. Li and Marshall [20] simulated the precipitation of particles on a single fiber by the CFD-DEM method, and then discussed the impacts of adhesion on particle deposition. Hund et al. [21] employed the CFD-DEM method to simulate the formation of particle bridges in solid-liquid separation, studied the impacts of particle concentration and feed flow rate on the formation of particle bridges, preliminarily revealed the formation mechanism of particle bridges, and verified the accuracy of the simulation results by comparing them with the experimental results. Additionally, Qian et al. [22,23] and Cao et al. [24] established a multi-layer three-dimensional fiber medium filtration model and simulated the flow and precipitation of particles in the model by using the CFD-DEM method. The results revealed that it was convenient and feasible to study the flow and precipitation of small particles in filter media by the CFD-DEM method, which were consistent with the experimental results. However, in these studies, the shape of the particles was specially processed and simplified to be spherical. Most of the particles applied in industrial production are non-spherical, and the shape greatly influences the dynamic behavior of the particles [25]. The ellipsoid models [26], hyper ellipsoid models [27], bonded sphere models [28], multi-sphere models [29], and polyhedron models [30] have been developed in DEM to describe the shape of particles more accurately to achieve a more realistic simulation. The most important difference between spherical particles and non-spherical particles is that the formers are prone to rolling under the same conditions when the CFD-DEM method is adopted [25]. However, with more accurate descriptions of the shape of the particles, the amount of calculation in simulation is increasing, especially in solving the movement of micron-sized particles. Rolling motion of the spherical particles is controlled by the rolling friction coefficient among particles and that between the particles and the membrane, so the coefficient can be introduced into the DEM to generate the rolling resistance. Therefore, spherical particles can replace the non-spherical particles by controlling the numerical range of the rolling friction coefficient. In this context, people began to replace non-spherical particles with spherical particles, and the rolling friction coefficient has been artificially increased to show some properties of non-spherical particles [31], aiming to satisfy the simulation requirements and reduce the amount of calculation.

There are many studies on the range of the rolling friction coefficient for spherical particles to replace non-spherical particles. Wensrich and Katterfeld [32] revealed that the rolling friction coefficient could indeed bring some properties of non-spherical particles to spherical particles. Xie et al. [25] proposed a method of replacing non-spherical particles with spherical particles based on the rolling friction and then studied the applicability of this method under different processes and operating conditions. The results indicated that the non-spherical particles can be replaced, to some extent, to reduce the amount of calculation and speed up the solution if a reasonable rolling friction coefficient can be applied. Xiong et al. [31] simulated particle deposition on a single fiber, studied the impacts of the rolling friction coefficient on the deposition morphology, coordination number, and porosity of particles, and then calibrated the rolling friction coefficient among particles and that between particles and fibers in fiber filtration, making the simulation results closer to the real observations. In general, the rolling friction coefficient significantly affects the movement of spherical particles. Therefore, correctly defining the rolling friction coefficient is the key to ensure that the simulation process and results are in line with reality.

Many researchers have noticed that spherical particles can be substituted for non-spherical particles by setting a proper rolling friction coefficient. However, calibration of the rolling friction coefficient is always the key- and difficult-point during the simplification. In this study, non-spherical lignin particles were simplified to spherical particles by applying the rolling friction coefficient to the latter. Effects of the rolling friction coefficient on the deposition of lignin particles in ceramic membranes were studied by the CFD-DEM coupling method at the single pore level. Firstly, the capture of lignin particles was demonstrated dynamically. Then, effects of the rolling friction coefficient on the morphology, coordination number distribution, and porosity of the lignin particles were investigated and analyzed. Finally, the rolling friction coefficient was calibrated. Introducing the correct rolling friction coefficient could simplify the DEM model and improve its accuracy, providing a more reasonable rolling friction coefficient for subsequent research on the more complex deposition of lignin particles in porous ceramic membranes.

## 2. Governing Equation of Fluid–Solid Two-Phase Flow

**Figure 1:**
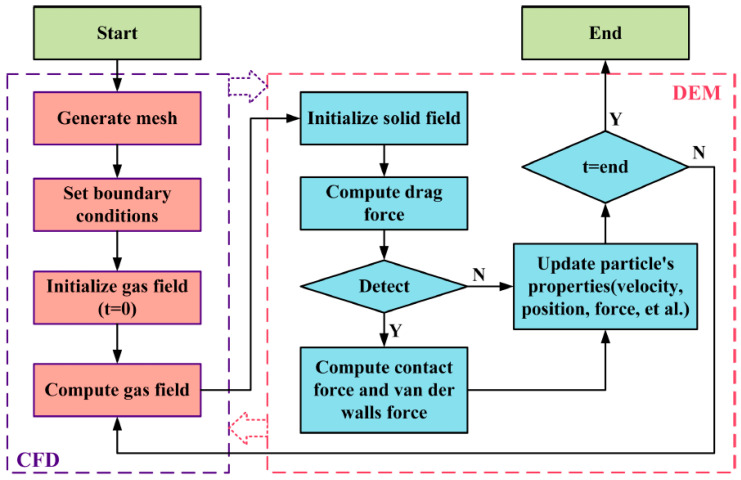
Flowchart of the CFD-DEM coupling.

The coupling solution process based on CFD-DEM was completed under the Euler-Lagrange coupling framework, in which the Navier-Stokes (N-S) control equation was adopted to solve the fluid movement based on the continuum assumption. In addition, the Newton’s Second Law was adopted to solve the movement of each particle, and the coupling between the two is realized by the Newton’s Third Law. The flowchart of coupling is shown in Figure 1 [16].

### 2.1. Fluid Phase Control Equation

In the coupling solution process, the particle phase greatly affected the movement characteristics of the fluid. Therefore, the void fraction was necessary to be incorporated into the traditional N-S equation to characterize the volume of the fluid phase in a specific computational grid. The control equation of the fluid phase can be expressed as follows [33,34]:

(1) $$\frac{\partial \rho \varphi }{\partial t}+\nabla \left(\rho \varphi u\right)=0$$

(2) $$\frac{\partial }{\partial t}\left(\rho \varphi u\right)+\nabla \left(\rho \varphi \vartheta u\right)=-\nabla \rho -S+\nabla \left(\vartheta \varphi \nabla u\right)+\rho \varphi g$$

(3) $$S=\sum_{i}^{n}F_{D}/∆V$$

where, ϑ, u, and ρ refer to the viscosity, velocity, and density of the fluid with the units of Pa·s, m/s, and kg/m^3^, respectively; *S* is the sum of the fluid resistance $F_{D}$ acting on the volume of a grid cell, N; and ∆V is the volume of the mesh unit, m^3^.

### 2.2. Discrete Model

During the movement of particles, the Newton’s Second Law was employed to solve the velocity and position of particles at each specific time based on the external force on particles. In this study, the external forces on particles included gravity, the interaction force between particles and fluid, the collision force among particles and that between particles and membranes, and the Van der Waals adhesion force among particles and that between particles and membranes. Therefore, the particle-phase control equation can be expressed as follows [14]:

(4) $$m_{p}\frac{du_{p}}{dt}=m_{p}g+F_{JKR}+F_{fp}+F_{c}$$

(5) $$I_{p}\frac{d\omega_{p}}{dt}=\sum_{i=1}^{k}\left(M_{i}+M_{JKR,i}\right)$$

where, $m_{p}$, $u_{p}$, $I_{p}$, $\omega_{p}$, and $M_{i}$, are mass, velocity, inertia moment, rotational speed, and collision torque of the particle, respectively, with the units of kg, m/s, kg·m^2^, rad/s, and N·m, respectively. $M_{JKR,i}$ is the adhesive torque in collisions with other particles, N·m; $F_{JKR}$ refers to the Van der Waals adhesion force, N; $F_{fp}$ is the particle-fluid force, N; and $F_{c}$ denotes the particle–particle contact force, N.

$F_{JKR}$ is the Van der Waals adhesion, which can be calculated by Equation (6) [35,36]:

(6) $$F_{JKR}=-4\sqrt{\pi \gamma E^{*}}\alpha^{3/2}+\frac{4E^{*}}{3R^{*}}\alpha^{3}$$

where, γ is the surface energy, J/m^2^; $E^{*}$ refers to the relative Young’s Modulus, Pa; α is the normal overlap, m; and $R^{*}$ represents the relative radius, m.

The equivalent Young’s Modulus $E^{*}$ and relative radius $R^{*}$ are defined as follows:

(7) $$\frac{1}{E^{*}}=\frac{\left(1-\upsilon_{i}^{2}\right)}{E_{i}}+\frac{\left(1-\upsilon_{j}^{2}\right)}{E_{j}}$$

(8) $$\frac{1}{R^{*}}=\frac{1}{R_{i}}+\frac{1}{R_{j}}$$

where, $E_{i}$ and $E_{j}$ are the Young’s Modulus of particle-*i* and particle-*j*, respectively, Pa; $\upsilon_{i}$ and $\upsilon_{j}$ are the Passion’s ratios of particle-*i* and particle-*j*, respectively; and $R_{i}$ and $R_{j}$ are radii of particle-*i* and particle-*j*, respectively, m.

$F_{fp}$ represents the force between the particle and fluid. In this study, the interaction force between liquid and solid phases only considers the drag force $F_{drag}$ and pressure gradient force $F_{p}$, so $F_{fp}$ can be expressed as Equation (9).

(9) $$F_{fp}=F_{drag}+F_{p}$$

$F_{drag}$ represents the fluid drag force of the particle, as shown in Equation (10).

(10) $$F_{drag}=0.5C_{D}\rho A\left|u-u_{p}\right|\left(u-u_{p}\right)$$

where, $F_{drag}$ is the drag force of the particle, N; *A* is the projection area of the particle, m^2^; $C_{D}$ refers to the drag coefficient, which depends on the Reynolds number Re, and can be calculated by Equation (11) [37].

(11) $$C_{D}=\left\{\begin{array}{c} \frac{24}{Re}\;\;\;\;\;\;\;\;\;\;\;\;\;\;\;\;\;\;\;\;\;\;\;\;\;\;\;\;\;\;\;\;\;\;Re\leq 0.5\\ \frac{24\left(1.0+0.25{Re}^{0.687}\right)}{Re}\;\;\;\;0.5<Re\leq 1000\\ 0.44\;\;\;\;\;\;\;\;\;\;\;\;\;\;\;\;\;\;\;\;\;\;\;\;\;\;\;\;\;\;\;\;\;\;Re>1000 \end{array}\right.,\;Re=\frac{\varphi \rho d_{p}\left|u-u_{p}\right|}{\mu }$$

$F_{p}$ is the pressure gradient force caused by the pressure gradient of particles moving in the flow field, as shown in Equation (12) [38].

(12) $$F_{p}=dp/dx=-\rho g-\rho udu/dx$$

$F_{c}$ is the particle–particle contact force, as shown in Equation (13) [39].

(13) $$F_{c}=F_{cn}+F_{ct}$$

where, $F_{cn}$ is the normal contact force acting on particles after particle collision, N, as expressed as Equation (14); and $F_{ct}$ is the tangential contact force acting on particles after particle collision, N, as written as Equation (15).

(14) $$F_{cn,ij}=\left(-k_{n}\alpha^{3/2}-c_{n}u_{ij}\cdot n\right)n$$

(15) $$F_{ct,ij}=-k_{t}\delta -c_{t}v_{ct}$$

where, $u_{ij}=u_{i}-u_{j}$ refers to the velocity of particle-*i* relative to particle-*j*, m/s; n is the unit vector pointing from the centroid of particle-*i* to the centroid of particle-*j*; δ represents the tangential displacement of contact point, m; and $v_{ct}$ denotes the sliding velocity vector.

### 2.3. The Directional Constant Torque Model

As mentioned in the first section, the most important difference between spherical and non-spherical particles during the movement is that the formers are easy to roll under the same conditions. Therefore, the principle of simplifying non-spherical particles into spherical particles can be described as follows. The rolling resistance is applied to the simplified spherical particles, so that they show the movement characteristics of non-spherical particles. In this case, they can replace the non-spherical particles. However, the rolling resistance is difficult to be determined in various industries. In the DEM, the directional constant torque model is the most widely accepted rolling resistance model, which can be calculated with Equation (16).

(16) $$M_{r}=-\mu F_{cn}R_{c}\omega_{c}$$

where, $M_{r}$ is the rolling friction resistance, N; μ represents the rolling friction coefficient; $R_{c}$ refers to the distance between the contact point and the center of the sphere, m; and $\omega_{c}$ is the angular velocity of the contact point, rad/s. Therefore, the determination of the rolling resistance can be converted to the determination of the rolling friction coefficient. Changing the rolling friction coefficient can alter the size of the rolling resistance, and the rolling friction coefficient can be calibrated through studying a series of physical quantities. In this way, the non-spherical particles can be replaced with the spherical particles.

## 3. Computational Set-Up

### 3.1. Geometry and Computational Domain

**Figure 2:**
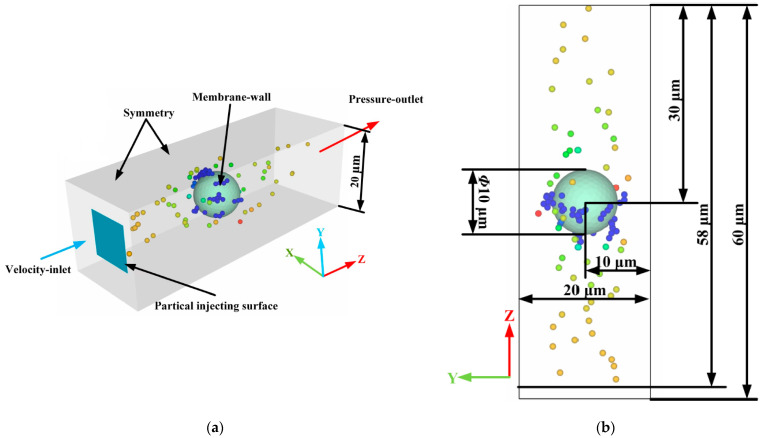
Pore structure of the ceramic membrane and the size of the computational domain. (**a**) Isometric view; (**b**) YZ plan view.

According to the microscopic characterizations of the structure and morphology, the filter channel in the ceramic membrane was formed by the accumulation of ceramic particles with a shape similar to a sphere. Such a porous structure for a ceramic membrane was difficult to model, and many membrane pores would negatively affect the ability to characterize the deposition morphology of particles in each membrane pore, making it difficult to calibrate the rolling friction coefficient. Therefore, the porous structure of the ceramic membrane was simplified in this study by analogy with the method introduced by Tao et al. [17] and Xiong et al. [31]. Figure 2 illustrated the simplified pore structure of a single ceramic membrane and the size of the computational domain. Among them, the X and Y directions of the computational domain size were twice the diameter of the ceramic particle, which exhibits similar results with those obtained by Xiong et al. [31] in the single fiber filtration model. The computational domain size along the direction of fluid flow is set to six times the diameter of the ceramic particle, according to the reports of Xiong et al. [31] and Qian et al. [23], to ensure the uniform flow of the black liquor and lignin particles in the inlet and outlet. Moreover, the shape of the ceramic particles was simplified accordingly. The ceramic particle spheres, similar to spheres, were simplified as spheres, and the rolling friction coefficient between the lignin particles and ceramic membranes was introduced in the solution setting. Calibrating the rolling friction coefficient between the lignin particles and ceramic membranes can make the physical model closer to reality and greatly accelerate the calculation.

### 3.2. Boundary Conditions and Parameter Settings

In this study, the EDEM 2020 and Fluent 2020R2 were coupled to calculate the particles’ movement and fluid fields through User-Defined Function (UDF). The coupling between CFD and DEM is implemented using the Eulerian coupling method, which considers the momentum exchange between liquid phase and particle phase, and the influence of particles relative to the liquid phase. Particles in the DEM are translated and rotated by the explicit time integral method, while the governing equation of fluid in the CFD is solved by the SIMPLE algorithm under the pressure base solver. The second order scheme is employed to disperse the pressure term, and the first order scheme discretizes the other terms. Meanwhile, the drag model of Ergun and Wen & Yu was selected. In addition, the black liquor adopted the speed import and pressure export. The lignin particles entered the flow field under the driving force of the black liquor. To ensure that the lignin particles entered the flow fields smoothly, the lignin particle generating surface was behind the velocity entrance. In consideration of the structural characteristics of the ceramic membrane, surfaces of the ceramic particles were set as the non-slip boundary condition, and the other boundaries of the computational domain were set as the symmetric boundary condition. The total calculation time was 2.2 ms, and the lignin particles were generated only within 0–2 ms, which ensured the complete deposition or flowing out of all the lignin particles in the computational domain at the end of the simulation. The main parameters used for simulation in this study are shown in Table 1 and Table 2.

**Figure 3:**
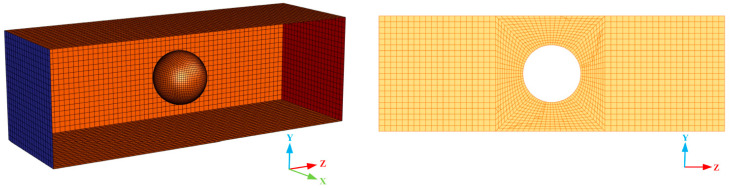
Mesh model of the fluid domain.

A semi-analytical CFD-DEM coupling interface was employed in this study. Instead of precisely analyzing the flow state around each particle, the semi-analytical interface redistributed the particle-phase volume spatially by introducing kernel functions and capturing the physical information of the background flow fields in the expanding region. The physical information in the background flow fields could be obtained reasonably and accurately no matter how many grids were covered. Therefore, the semi-analytical interface could simulate the movement of particles in a fluid grid that is equivalent to or even slightly smaller than the size of particles. It solved the problem that the unresolved CFD-DEM coupling interface cannot be applied to the field of ceramic membrane filtration, and solved the problem that the resolved CFD-DEM coupling interface had a large amount of calculation. Therefore, the size of the fluid domain grid was set within a range equal to or slightly smaller than the diameter of the lignin particle in this study. In this case, the grid independence was verified based on the clean filtration stage, as displayed in Table 3. The semi-analytical CFD-DEM coupling interface exhibited good stability, and the simulation results could be correctly represented when the number of grids was 46,400. Figure 3 demonstrated the fluid domain grid model when the number of grids was 46,400. As it illustrated, the fluid domain was meshed by ICEM CFD, and the hexahedral mesh was generated by the block topology function under the ICEM CFD. The minimum and maximum mesh sizes were 0.2 μm and 0.7 μm, respectively, and the maximum mesh mass was 1. It should be noted that the grid quality here was measured by the determinant with a value range of 0–1. The more the value was close to 1, the more perfect the grid was.

## 4. Results and Discussion

### 4.1. Particle Deposition Process

**Figure 4:**
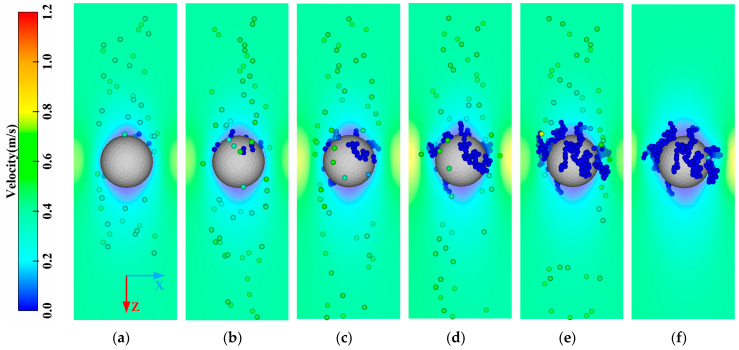
Change in the deposition morphology of the lignin particles with time. (**a**) A (T = 0.1 ms); (**b**) B (T = 0.5 ms); (**c**) C (T = 1.0 ms); (**d**) D (T = 1.5 ms); (**e**) E (T = 2.0 ms); (**f**) F (T = 2.2 ms).

**Figure 5:**
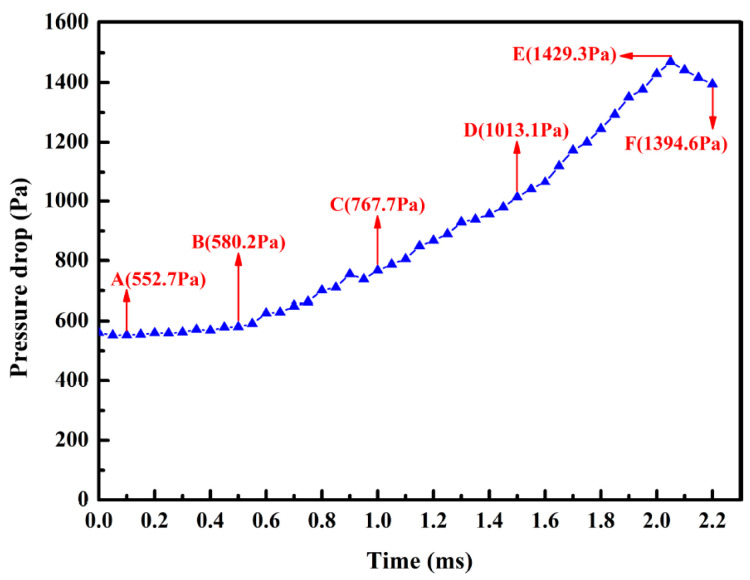
Pressure drop curve with time.

Figure 4 illustrates the deposition process of the lignin particles with the rolling friction coefficient among the lignin particles of $\mu_{p-p}=1.2$, and that between the lignin particles and membranes of $\mu_{p-m}=1.0$. The deposition process of the lignin particles could be roughly divided into two stages: the capture of the ceramic membrane (A–B), and the capture of the deposited lignin particles (B–F). In the initial stage of filtration (A–B), the lignin particles were mainly captured and approximately evenly distributed on the upstream side surface of the ceramic membrane. At this stage, the number of lignin particles captured was not large, and the pressure drop increased slightly. As indicated by the pressure drop curve in the A–B stage (Figure 5), the pressure drop increased linearly but the growth rate was relatively slow. As the filtration process proceeded (B–F), the lignin particles began to be captured by the lignin particles deposited, leading to a significant change in the deposition morphology. The deposited lignin particles began to grow outward, forming a dendritic structure, which became more obvious and independent as the filtration process progressed. Finally, the deposition morphology of the lignin particles showed the shape of a “forest.” At this stage, most of the lignin particles were captured by the dendritic structure, greatly increasing the number of captured particles and the pressure drop. As demonstrated in the pressure drop curve in the B–F stage in Figure 5, the slope of the pressure drop curve with time gradually increased, indicating that the efficiency in capturing lignin particles was constantly increasing. However, it should be noted that the lignin particles were only generated within 0–2 ms, so the pressure drop decreased gradually when the filtration time was longer than 2 ms (E–F stage in Figure 5).

**Figure 6:**
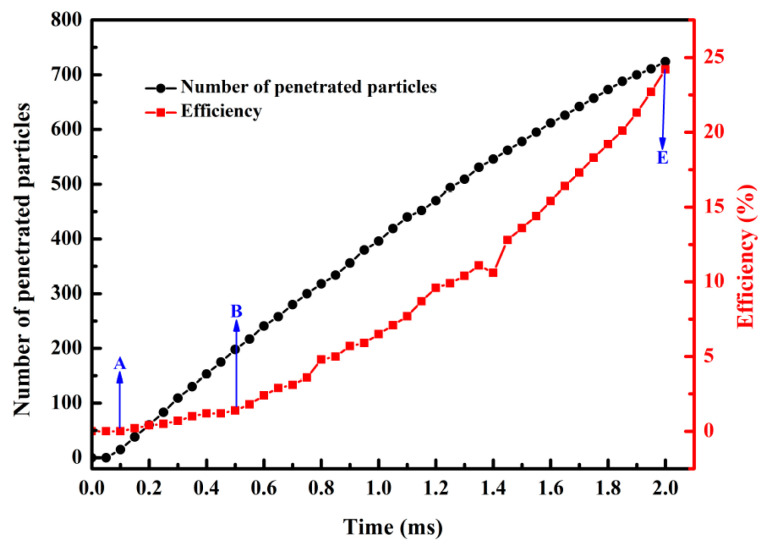
Curves of the cumulative number of penetrated particles and efficiency in capturing lignin particles with time.

The cumulative number of penetrating particles and the efficiency in capturing lignin particles during filtration were counted and calculated to further investigate the impacts of dendritic structure growth on the efficiency in capturing lignin particles. As demonstrated in Figure 6, the cumulative number of penetrating particles and the efficiency in capturing lignin particles both increased as the filtration time increased. However, the cumulative number of penetrated particles grew slowly, and the efficiency in capturing lignin particles exhibited a faster growth rate. This is because the deposited lignin particles formed dendritic structures, exerting a secondary trapping effect on the lignin particles, so that an increasing number of lignin particles were captured, and the retention rate was increased. In other words, formation of the dendritic structure significantly affected the efficiency in capturing lignin particles.

### 4.2. Impacts of the Rolling Friction Coefficient on Deposition Morphology of Lignin Particles

The actual lignin particles had greater rolling friction resistance than the ideal spherical lignin particles due to their shapes, thereby limiting the movement among lignin particles, and that between lignin particles and membranes [31]. Previous studies have shown that the deposition morphology of lignin particles was correlated with the external force. When the rolling friction resistance was not enough to resist the external force, the lignin particles would move until they reached a new equilibrium state [40], which significantly affected the deposition morphology of the lignin particles. Therefore, effects of the rolling friction coefficient on the deposition morphology of lignin particles were quantitatively characterized when the rolling friction coefficient among the lignin particles was $\mu_{p-p}=0-3$, and that between the lignin particles and membranes was $\mu_{p-m}=0-3$.

**Figure 7:**
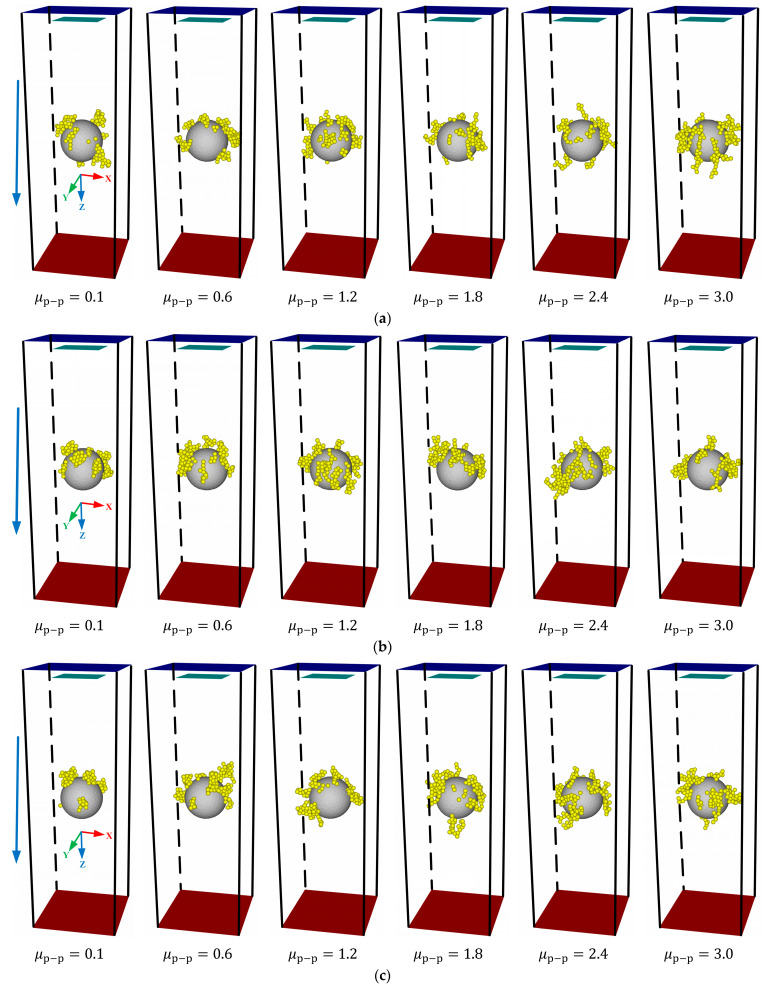

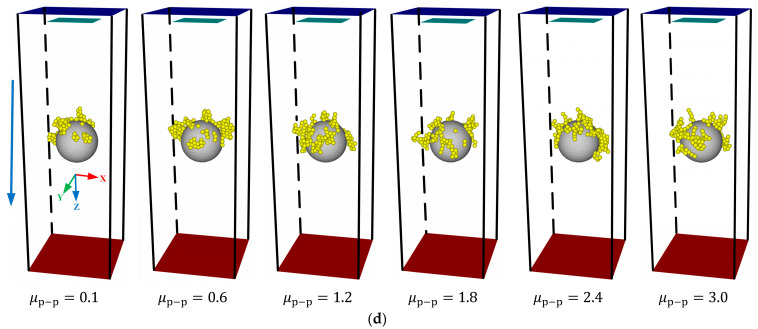
Impacts of the rolling friction coefficient on the deposition morphology of lignin particles. (**a**) $\mu_{p-m}=0.1$; (**b**) $\mu_{p-m}=1.0$; (**c**) $\mu_{p-m}=2.0$; (**d**) $\mu_{p-m}=3.0$.

Figure 7 shows the deposition morphology of lignin particles under different rolling friction coefficients. It revealed that regardless of $\mu_{p-m}$, the deposition morphology of lignin particles showed a strong regularity with increasing $\mu_{p-p}$. When $\mu_{p-p}\leq 0.6$, the particles were deposited on the surface of the ceramic membrane in the form of agglomeration, and no dendritic structure was observed, which was inconsistent with the experimental results of Huang et al. [41]. The reason is that the lignin particles were easier to roll because of a too-small rolling resistance, and the dendritic structure formed at the early stage lodged down to the ceramic membrane surface so that the deposition morphology of lignin particles exhibited an agglomeration. As the rolling friction coefficient among lignin particles increased, the corresponding rolling resistance increased, and the lignin particles were difficult to rotate around the contact point. At this moment, an obvious dendritic structure was observed. As there was an increase in the rolling friction coefficient among the lignin particles, the dendritic structure became increasingly obvious and independent, making the deposition morphology of the lignin particles look like a “forest.” This finding is consistent with the deposition morphology of the particles on the single fiber observed by Huang et al. [41].

Moreover, Figure 7 discloses that the rolling friction coefficient between the lignin particles and membranes had no obvious effect on the deposition morphology of the lignin particles. This might be because the number of contacts among the lignin particles was much larger than that between the lignin particles and membranes [31]. Table 4 showed the proportion of the number of contacts between the lignin particles and membranes to the total number of contacts under different rolling friction coefficients. The number of contacts between the lignin particles and membranes accounted for only 18.4% at most, weakening the effects of the rolling friction coefficient between them on the deposition morphology of lignin particles.

### 4.3. Impacts of the Rolling Friction Coefficient on the Deposition Structure of Lignin Particles

Generally, coordination number (*Q*) and porosity (ε) are two important parameters describing the structural properties of particle packing [31]. The former refers to the number of particles in contact with the central particles, and its size reflects the agglomeration properties of the stacking structure. The latter reflects the compactness of the accumulation structure. Therefore, this section discusses the effects of the rolling friction coefficient on the average coordination number, coordination number distribution, and porosity of lignin particles.

**Figure 8:**
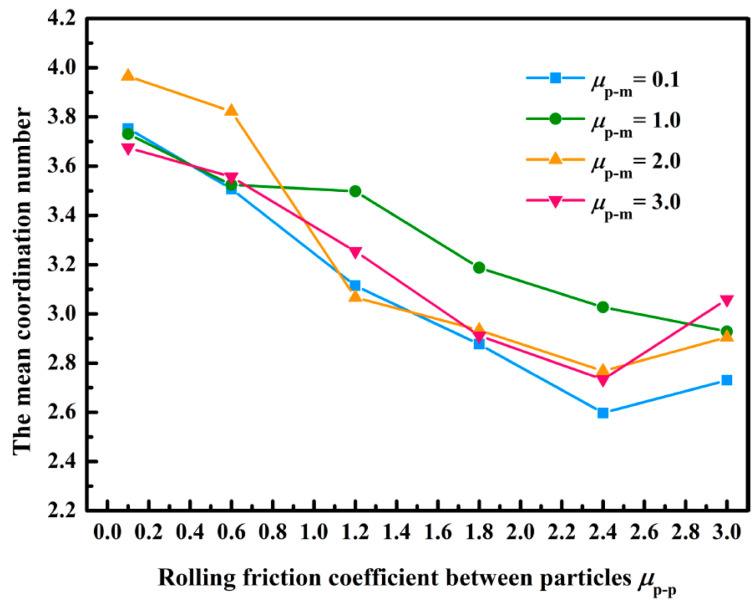
Impacts of the rolling friction coefficient on average coordination number.

Figure 8 displays the curve of the average coordination number of lignin particles with the rolling friction coefficient. It illustrated that the average coordination number decreased with the increasing rolling friction coefficient among the lignin particles, and the change law was consistent with the results obtained by Xiong et al. [31]. When the rolling friction coefficient among the lignin particles increased from 0.1 to 3.0, the average coordination number decreased from 3.96 to 2.73; it indicated that the number of contacts among the lignin particles gradually decreased, and the dendritic structure became more independent as the rolling friction coefficient among the lignin particles increased. Such a conclusion was consistent with the change law of the deposition morphology of lignin particles mentioned in Section 4.2. Besides, the rolling friction coefficient between the lignin particles and membranes slightly affected the average coordination number, which might be because the number of contacts between the lignin particles and membranes was too small (as described in Section 4.2). Yang et al. analyzed the stacking structure of settling particles, only under the action of gravity, and revealed that the average coordination number of particles at 1 μm was 2.13 [42]. However, the fluid force would increase the average coordination number, so the reasonable and acceptable minimum average coordination number was determined as 2.13 in this study; that is, the rolling friction coefficient in this simulation range could meet the requirements of the coordination number.

**Figure 9:**
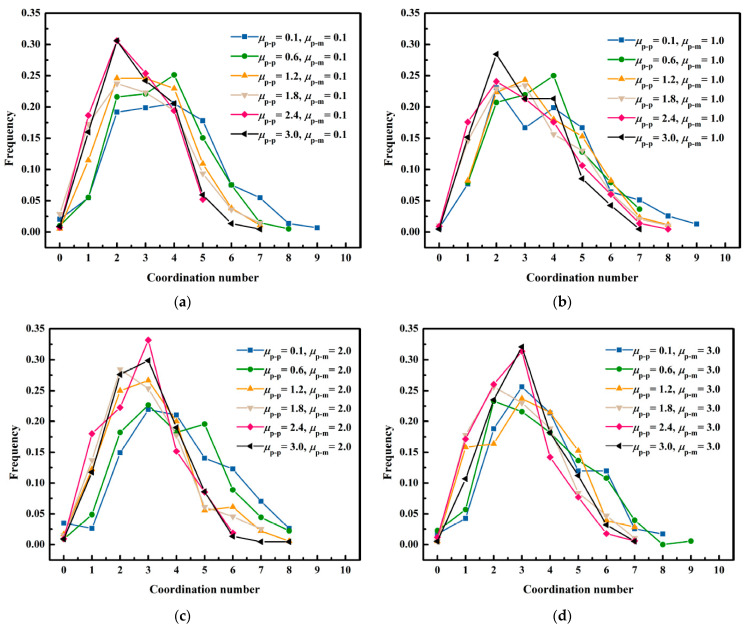
Impacts of the rolling friction coefficient among the lignin particles on coordination number distribution. (**a**) $\mu_{p-m}=0.1$; (**b**) $\mu_{p-m}=1.0$; (**c**) $\mu_{p-m}=2.0$; (**d**) $\mu_{p-m}=3.0$.

Figure 9 describes the impacts of the rolling friction coefficient among the lignin particles on the coordination number distribution. The increase in the rolling friction coefficient made the dendritic structure formed by the deposition of lignin particles more independent, decreasing the distribution range of the coordination number with an increase in the rolling friction coefficient among the lignin particles. The research conducted by Yang et al. suggested that particles at 1 μm were only subject to the action of gravity, and the distribution range of the coordination number was mainly 1–4 [42]. However, the contact among different particles became closer due to the action of fluid force, which expanded the distribution range of the coordination number.

**Figure 10:**
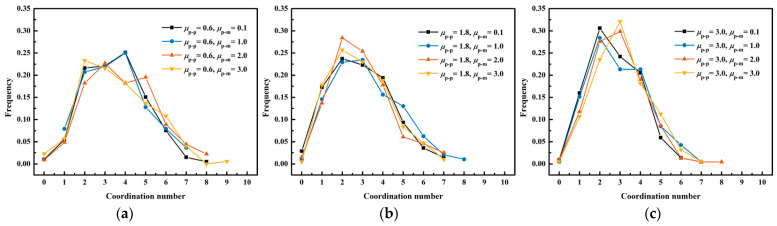
Impacts of the rolling friction coefficient between the lignin particles and membranes on the coordination number distribution (**a**) $\mu_{p-p}=0.6$; (**b**) $\mu_{p-p}=1.8$; (**c**) $\mu_{p-p}=3.0$.

Figure 10 exhibits the impacts of the rolling friction coefficient between the lignin particles and membranes on the coordination number distribution. It illustrates that the rolling friction coefficient between the lignin particles and membranes did not affect the coordination number distribution. Therefore, it can be concluded that the rolling friction coefficient among the lignin particles was the primary factor affecting the coordination number distribution.

The porosity of the particle packing structure can be calculated by Equation (17) [31]:

(17) $$\varepsilon =1-{\left[\left(\overset{-}{Q}/\overset{-}{Q_{0}}-1\right)/\left(m-n\right)\right]}^{1/4}$$

where, $\overset{-}{Q}$ is the mean coordination number; $\overset{-}{Q_{0}}$ = 2.02; m = 87.38; and n = 25.81.

**Figure 11:**
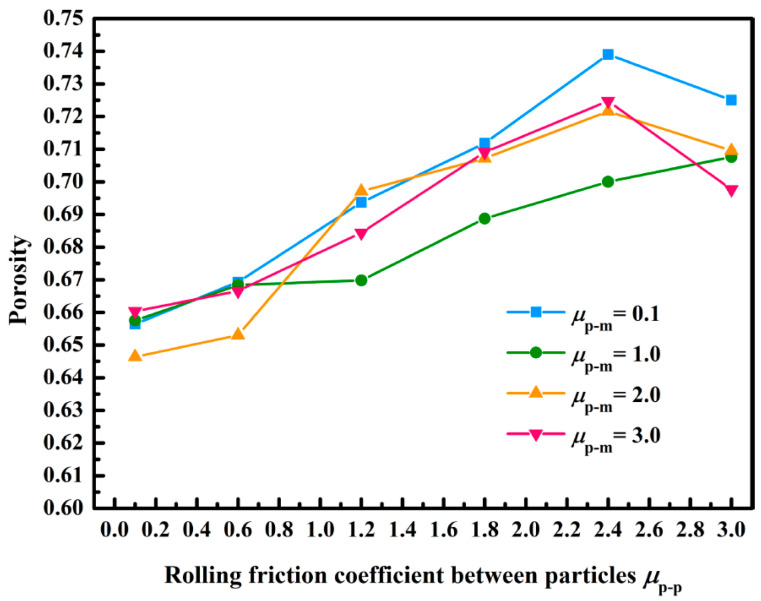
Impacts of the rolling friction coefficient on porosity.

Figure 11 displays the variation curve of porosity with the rolling friction coefficient. As the rolling friction coefficient between the lignin particles and membranes did not affect the average coordination number, it also did not affect the porosity [31]. We observed that the porosity increased from 0.65 to 0.73 when the rolling friction coefficient among the lignin particles increased from 0.1 to 3.0. This was because the increase in the rolling friction coefficient between the lignin particles improved the rolling resistance among the lignin particles, which enhanced the interaction among the lignin particles, thus increasing the porosity. According to the experimental results of Dingwell et al., porosity of the filter cake formed by the lignin particles was 0.61–0.71 [43]. When the rolling friction coefficient among the lignin particles was 0.1–2.4, the porosity was 0.65–0.72. Therefore, it was more in line with the actual porosity when the rolling friction coefficient among the lignin particles was set to 0.1–2.4 in the simulation, according to the experimental results of Dingwell et al.

## 5. Conclusions

In this study, a method of simplifying the shape of lignin particles into spheres was proposed to solve the difficult CFD-DEM solution and low computational efficiency of non-spherical lignin particles. Then, the CFD-DEM method was adopted to simulate the deposition of spherical lignin particles in the pores of a single ceramic membrane. Later, impacts of the rolling friction coefficient on the deposition morphology, average coordination number, coordination number distribution, and porosity of lignin particles were investigated and analyzed. In addition, the rolling friction coefficient was calibrated. Finally, the following conclusions were drawn:

(1) The deposition of lignin particles on ceramic membranes was dynamic, which mainly included capturing ceramic membranes in the initial filtration and deposited lignin particles. Formation of a dendritic structure not only made the deposition morphology of lignin particles look like a “forest,” but also greatly improved the efficiency in capturing the lignin particles.
(2) The rolling friction coefficient among the lignin particles crucially affected the deposition morphology, average coordination number, coordination number distribution, and porosity of the particles; the average coordination number decreased from 3.96 to 2.73, and the porosity increased from 0.65 to 0.73, when it increased from 0.1 to 3.0.
(3) Reasonably providing a rolling friction coefficient among the lignin particles could replace spherical lignin particles with non-spherical particles. Impacts of the rolling friction coefficient on the deposition morphology, coordination number, and porosity of lignin particles enabled the simulation to be closer to the real lignin filtration by setting the rolling friction coefficient among the lignin particles as 0.6–2.4.

## Figures and Tables

**Table 1:** Physical parameters.

Material	Particle	Membrane	Black Liquor
Diameter (μm)	1	10	-
Density (kg/m^3^)	1451	3100	1004
Shear modulus (Pa)	2 × 10^7^	7 × 10^10^	-
Poisson’ ratio	0.25	0.2	-
Viscosity (Pa·s)	-	-	1.467
Velocity (m/s)	0.5	-	0.5

**Table 2:** Collision and simulation parameters [17,23,31].

**Collision Parameters**	**Coefficient of Restitution**	**Coefficient of Static Friction**	**Coefficient of Rolling Friction**	**Surface Energy (J/m^2^)**
Particle–particle	0.1	2.0	0.1–3	0.6
Particle–membrane	0.1	2.0	0.1–3	1
**Simulation Parameters**	**Particle Generation Rate/s**	**Total Number of Particles**	**Time Step of DEM/s**	**Time Step of CFD/s**
	5 × 10^5^	1000	1 × 10^−10^	1 × 10^−8^

**Table 3:** Mesh independence verification.

Group	Mesh Quantity	Pressure Drop (Pa)
1	28,900	527.34924
2	37,544	527.9762
3	46,400	528.85345
4	50,270	529.08069
5	59,048	529.62501

**Table 4:** Proportion of the number of contacts between the lignin particles and membranes to the total.

	$\mu_{p-m}$	0.1	1.0	2.0	3.0
$\mu_{p-p}$					
0.1		13.8%	17.3%	13.7%	17.3%
0.6		12.1%	11.1%	10.4%	11.3%
1.2		16.7%	11.2%	14.6%	15.5%
1.8		18.4%	12.8%	13.0%	14.2%
2.4		17.5%	15.1%	14.4%	16.3%
3.0		17.2%	14.2%	16.4%	13.1%

## Data Availability

Not applicable.
